# Evaluation of *TaMFT-3A* and *TaMKK3-4A* alleles on wheat pre-harvest sprouting

**DOI:** 10.3389/fpls.2025.1594385

**Published:** 2025-05-14

**Authors:** Bo-Wen Zhang, Bai-Song Yang, Xiao-Neng Wan, Xin Ma, Kai-Di Lyu, Han Wang, Shu-Ying Yang, Hui-Hui Zhang, Shu-Nv Hao, Jian Ma, Guo-Zhong Sun

**Affiliations:** ^1^ College of Agronomy, Jilin Agricultural University, Changchun, China; ^2^ National Engineering Research Center for Crop Molecular Breeding, Institute of Crop Sciences, Chinese Academy of Agricultural Sciences (CAAS), Beijing, China

**Keywords:** wheat, pre-harvest spouting, recombinant inbred line, haplotype, marker-assisted selection

## Abstract

Pre‐harvest sprouting (PHS) is a significant challenge affecting global production of wheat (*Triticum aestivum* L.). Resistance to PHS is governed by both genetic and environmental factors, making reliable molecular markers essential for enhancing PHS resistance through molecular marker‐assisted selection (MAS). Genes *TaMFT-3A* and *TaMKK3-4A* have been cloned in wheat and are known to regulate PHS resistance in white-grained varieties. In this study, we assessed the allelic variations in these genes and their combined effects on PHS resistance using two recombinant inbred line (RIL) populations, Wanxianbaimaizi/Zhongyou 9507 (WZ) and Wanxianbaimaizi/Jing 411 (WJ), under two distinct field environmental conditions. PHS resistance was assessed by measuring seed germination in physiologically mature stage, correlation and ANOVA were used to analyze PHS data. The germination percentage (GP) and germination index (GI) were significantly correlated across both RIL populations. Specific allelic variations at positions -222, +646, and +666 in the *TaMFT-3A* gene strongly correlated with PHS resistance. The CGA haplotype at these loci was linked to the highest resistance, while the TAA haplotype was associated with the lowest resistance levels. Additionally, haplotype variation at the +660 locus of *TaMKK3-4A* demonstrated a weak but environmentally modulated correlation with PHS resistance. This study provides a theoretical foundation for utilizing *TaMFT-3A* and *TaMKK3-4A* in molecular breeding strategies to enhance wheat resilience to PHS.

## 1 Introduction

Wheat (*Triticum aestivum* L.) is a crucial staple crop, providing approximately 20% of the protein and calories consumed worldwide, and feeds nearly 40% of the global population (FAO, 2023). Pre-harvest sprouting (PHS) is the phenomenon in which cereal grains germinate on the ear before harvest due to wet weather. The global economic losses due to PHS exceed $1 billion annually (Black et al., 2006). In China, PHS affects approximately 83% of wheat growing areas (Xiao et al., 2002). In the years 2016, 2018 and 2023, significant incidents of wheat PHS occurred across several provinces, including Jiangsu, Anhui, Sichuan, Hubei and Henan of China (Dong et al., 2024). PHS not only reduces yield but also adversely affects quality, particularly processing quality. In severe cases, seeds may lose viability for sowing (Lang et al., 2021; Chang et al., 2023).

The resistance of wheat to PHS is a complex quantitative trait regulated by multiple genes and is significantly influenced by environmental factors. This complexity complicates its management in breeding practices, PHS remains a persistent problem in triticale and in other cereal crops (Sun et al., 2003; Moullet et al., 2022). Therefore, evaluating the effectiveness of molecular markers associated with PHS resistance genes in wheat breeding lines is essential for enhancing resistance through marker-assisted selection (MAS). Introduction of PHS resistance genes have lead to two new agronomically performant and PHS-resistant triticale cultivars (Moullet et al., 2022).

Currently, quantitative trait loci (QTLs) related to PHS resistance are mapped across all wheat chromosomes (Tai et al., 2021). Several genes associated with seed dormancy have been cloned, including *TaMFT-3A* (Shingo et al., 2011; Liu et al., 2015), *TaMKK3-4A* (Torada et al., 2016; Martinez et al., 2020), and *Tamyb10* (Lang et al., 2021). The MOTHER of FLOWERING TIME (*MFT*) gene regulates seed dormancy in Arabidopsis thaliana by modulating the levels of abscisic acid (ABA) and gibberellin (GA) (Xi et al., 2010; Barua et al., 2012). Within the wheat *TaMFT-3A* gene, several allelic variations are associated with PHS resistance. One single nucleotide polymorphism (SNP) (–222) located in the promoter region enhances seed dormancy, whereas two SNPs (+646 and +666) within the coding region reduce seed dormancy by creating a mis-splicing site and introducing a premature stop codon, respectively (Liu et al., 2015). Additionally, an insertion or deletion of 33 bp at the –194 bp position within the promoter is closely correlated with PHS resistance (Jiang et al., 2018). Lei et al. (2013) identified a 12 bp insertion in the coding region of *TaMFT-3A* for PHS resistance. Gene *TaMKK3-4A* encodes a mitogen‐activated protein kinase that plays a crucial role in protein phosphorylation modifications within the ABA signaling pathway (Torada et al., 2016; Chang et al., 2022). Two SNP alleles, C660R and A660S at position 660, as well as A1093R and G1093S at position 1093 in the coding region of *TaMKK3-4A*, are linked to PHS resistance (Shorinola et al., 2017; Martinez et al., 2020). Various combinations of allelic variations in *TaMFT-3A* and *TaMKK3-4A* are closely correlated with PHS resistance levels in wheat. The SNPs (–222), (+646), and (+666) in *TaMFT-3A* occurring in a CGA combination resulted in optimal PHS resistance (Wang et al., 2020). Conversely, a TAT combination results in the highest germination rate. Although *TaMFT-3A* can significantly enhance PHS resistance, its effectiveness varies depending on environmental conditions and genetic background (Lin et al., 2018). Similarly, the *TaMKK3-4A* gene also significantly reduced PHS but its effectiveness was also influenced by environments (Lin et al., 2018). In contrast, no association between allelic variations at *TaMKK3-4A* and PHS resistance was found within a hard red winter wheat doubled haploid population from North America (Vetch et al., 2022).

Consequently, the impact of the interaction between *TaMFT-3A* and *TaMKK3-4A* on the regulation of PHS resistance in wheat breeding requires further evaluation. This study aims to assess the germination index (GI) and germination percentage (GP) of two recombinant inbred line (RIL) populations, Wanxianbaimaizi/Zhongyou 9507 (WZ) and Wanxianbaimaizi/Jing 411 (WJ), across two distinct environments. Subsequently, we utilized the two RILs, which possess different alleles of *TaMFT-3A* and *TaMKK3-4A*, to examine the individual and combined genetic effects of these alleles on PHS resistance. This research will provide valuable insights for the marker-assisted breeding of wheat varieties with enhanced PHS resistance.

## 2 Materials and methods

### 2.1 Plant materials

The WZ population (103 F_10_ RIL) was generated from the cross between the resistant Wanxianbaimaizi (white-grained landrace) and the susceptible winter wheat cultivar `Zhongyou 9507` (Li et al., 2021), the WJ population (176 F_10_ RIL) was generated from the cross between Wanxianbaimaizi and the susceptible winter wheat cultivar `Jing 411` (Zhu et al., 2019). Winter wheat cultivars Henong 6049, Jimai 20, Aikang 58 (Wang et al., 2020), Cranbrook, and Halberd (Shorinola et al., 2017), which carry different alleles of genes *TaMFT-3A* and *TaMKK3-4A* genes, were used as the controls (**Table 1**).

**Table 1:** Function markers associated with wheat PHS and control varieties.

Gene	Primer	Sequence (5’-3’)	Haplotype	GI	Varieties
*TaMFT-3A*	PHS1-222-C	TCACGCATCAGCGATCGAC	C	L	Henong 6049
	PHS1-222-T	TCACGCATCAGCGATCGAT	T	H	Jimai 20
	PHS1-222-R	GCTTACGCTAAGCAGGTGGCTA			
	PHS1-646-G	GGTGGAACAGATGCAACTAAAGG	G	L	Jimai 20
	PHS1-646-A	GGTGGAACAGATGCAACTAAAGA	A	H	Aikang 58
	PHS1-646-R	GTGAGTGTTATATGAAACTAATGATCCATT			
	PHS1-666-T	GTGAGTGTTATATGAAACTAATGATCCATTT	T	L	Aikang 58
	PHS1-666-A	GTGAGTGTTATATGAAACTAATGATCCATTA	A	H	Jimai 20
	PHS1-666-R	ACCGGGTGGAACAGATGCAACTAAA			
*TaMKK3-4A*	TaMKK3-4A-snp1-G	TTTTGCTTCGCCCTTAAGG	G	L	Cranbrook
	TaMKK3-4A-snp1-T	TTTTGCTTCGCCCTTAAGT	T	H	Halberd
	TaMKK3-4A-snp1-R	GCATAGAGATCTAAAGCCAGCA			

H means high GI, L means low GI.

### 2.2 Planting and seed germination test

During the 2020–2021 growing season, Wanxianbaimaizi, Zhongyou 9507, and Jing 411, as well as the corresponding RIL populations, were grown in Beijing (116.30′N, 39.96′E) and in Gaocheng, Hebei Province (114.84′N, 38.02′E). Each plant entry was sown in a single row 2 m in length and a row spacing of 25 cm with 50 seeds per row and two replicates per field trial. Conventional field management and pest control measures were implemented. The flowering stage was recorded in the field according to Zadoks scale (Zadoks et al., 1974), and physiologically mature spikes were harvested about 35 days post-anthesis (Zadoks scale 91). The two environmental conditions listed in **Supplementary Table S1** from heading (Zadoks scale 50) to ripening (Zadoks scale 94). The harvested spikes were air-dried for three days and subsequently threshed. The seeds were then stored at -20°C to maintain dormancy until germination tests were conducted.

For the germination assay, 50 uniformly-sized seeds were selected for each material. The seeds were placed in Petri dishes lined with double-layer filter paper and moistened with sterile distilled water. Three replicates were prepared for each material. The seeds were incubated in the dark at 22°C for three days, during which the filter paper was kept moist. Photographs were taken at the same time each day to document germination progress. Germination was defined as the emergence of the radicle through the seed coat. The number of germinated seeds per day was recorded using ImageJ software (https://imagej.net/ij/). The GI and GP were calculated according to the following formula (Chang et al., 2010; Kader, 2005):


GI={(3×n1+2×n2+1×n3)/(3×N)}×100%

GP=(Final number of seeds germinated in three days)/N×100%

where n1, n2, and n3 represent the number of germinated seeds on the 1st d, 2nd d, and 3rd d, respectively, and N is the total number of seeds tested.

### 2.3 Detection of *TaMFT-3A* and *TaMKK3-4A* alleles by KASP markers

Plants were initially grown in pots. At the three-leaf stage, leaves from five seedlings per entry were pooled in equal amounts for DNA extraction using the Plant Genome DNA Extraction Kit (Tiangen Biochemical Technology Co., Ltd., Beijing, China). KASP markers specific to the allelic variants of the *TaMFT-3A* and *TaMKK3-4A* genes, along with additional base sequences for the FAM (GAAGGTGACCAAGTTCATGCT) and HEX (GAAGGTCGGAGTCAACGGATT) fluorescent lables, were synthesized (**Table 1**). The reaction mixture was prepared using the KASP kit (LGC, Middlesex, UK) and comprised 0.112 μL KASP Assay Mix (100 μmolL⁻¹), 4.48 μL of 2× KASP Master Mix (containing Mg²^+^;), 4.4 μL template DNA (approximately 50ng μL⁻¹), and ddH_2_O to a final volume of 10μL. The KASP reaction protocol was as follows: an initial pre-denaturation at 94°C for 15 min; then 10 cycles of 94°C for 20 s followed by annealing for 60 s, with the annealing temperature decreasing from 61°C to 55°C (a decrement of 0.6°C per cycle); and finally 26–30 cycles of 94°C for 20 s and 55°C for 60 s. Fluorescent signals were detected using the Omega SNP typing instrument (BMG LABTECH, Ortenberg, Germany), and genotypes were determined using the KlusterCaller™ (LGC, Middlesex, UK).

### 2.4 Data analysis

Microsoft Excel was employed to calculate the mean values, standard deviations, and distribution frequencies of GI and GP across the two environments. Statistical analyses, including correlation analyses between GI and GP and one-way analysis of variance (ANOVA) for GI differences between environments, were conducted using SPSS (https://spssau.com/). A *t*-test was applied to assess significant differences in GI among different allelic variations at positions –222, +646, and +666 of the *TaMFT-3A* gene and at position +660 of the *TaMKK3-4A* gene across the two environments. Additionally, *t*-test was used to evaluate significant differences in GI among various allelic variation combinations of these PHS resistance gene loci between the two environments.

## 3 Results

### 3.1 Allelic variations of *TaMFT-3A* and *TaMKK3-4A* genes in parents and evaluation of PHS resistance

**Figure 1:**
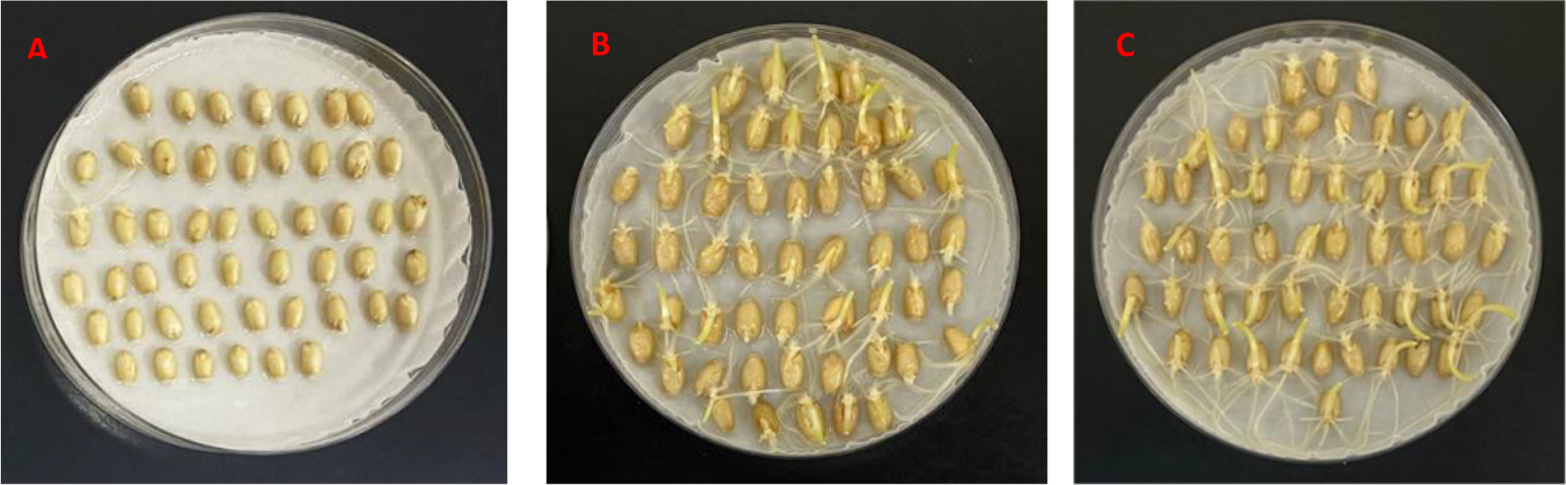
Germination of seeds harvested from site Beijing after three days for Wanxianbaimaizi **(A)**, Zhongyou 9507 **(B)**, and Jing 411 **(C)**.

The average GI values of Wanxianbaimaizi in Gaocheng and Beijing sites were lower than those of Zhongyou 9507 and Jing 411 (**Figure 1**), with the differences reaching an extremely significant level (**Table 2**). Regarding the allelic variations in the *TaMFT-3A* and *TaMKK3-4A* genes, Wanxianbaimaizi exhibited the nucleotides C, G, and T at positions –222, +646, and +666 of *TaMFT-3A*, respectively; G at position +660 of *TaMKK3-4A*. Collectively, these four loci constitute the PHS-resistant haplotype (Lei et al., 2013; Wang et al., 2020). In contrast, both Zhongyou 9507 and Jing 411 carried T, A, and A at positions –222, +646, and +666 of *TaMFT-3A*, respectively, and T at position +660 of *TaMKK3-4A*, representing the PHS-susceptible haplotype (Martinez et al., 2020; Wang et al., 2020).

**Table 2:** Alleles of PHS resistance genes and germination index for the parents of the RIL populations.

Parents	TaMFT-3A			TaMKK3-4A	GI (%)
	-222	+646	+666	+660	
Wanxianbaimaizi	C	G	T	G	17.01 ± 0.00
Zhongyou 9507	T	A	A	T	85.16 ± 14.88^**^
Jing 411	T	A	A	T	83.39 ± 1.24^**^

GI is the average between two environments, expressed as Mean ± SD. **Significance at *P* < 0.01.

### 3.2 Analysis of PHS resistance of two RIL population

In the WZ RIL population, the Pearson correlation coefficients between GP and GI were 0.832 in Beijing and 0.980 in Gaocheng. In the WJ RIL population, the correlation was 0.941 in Beijing and 0.888 in Gaocheng (**Table 3**). These results indicate a positive correlation between GP and GI in both RIL populations under the two environmental conditions (*P* < 0.01).

**Table 3:** Correlation analysis of GP and GI of the two RILs in two environments.

RILs	Environment	GI (%)	GP (%)	Correlation coefficient (GI vs. GP)
WZ	Beijing	53.59 ± 17.81	79.58 ± 17.93	0.832**
	Gaocheng	53.21 ± 17.96	78.50 ± 20.08	0.980**
WJ	Beijing	48.55 ± 18.69	71.26 ± 21.13	0.941**
	Gaocheng	59.47 ± 21.59	95.12 ± 6.89	0.888**

Data are expressed as mean ± SD. **Significance at *P* < 0.01.

**Figure 2:**
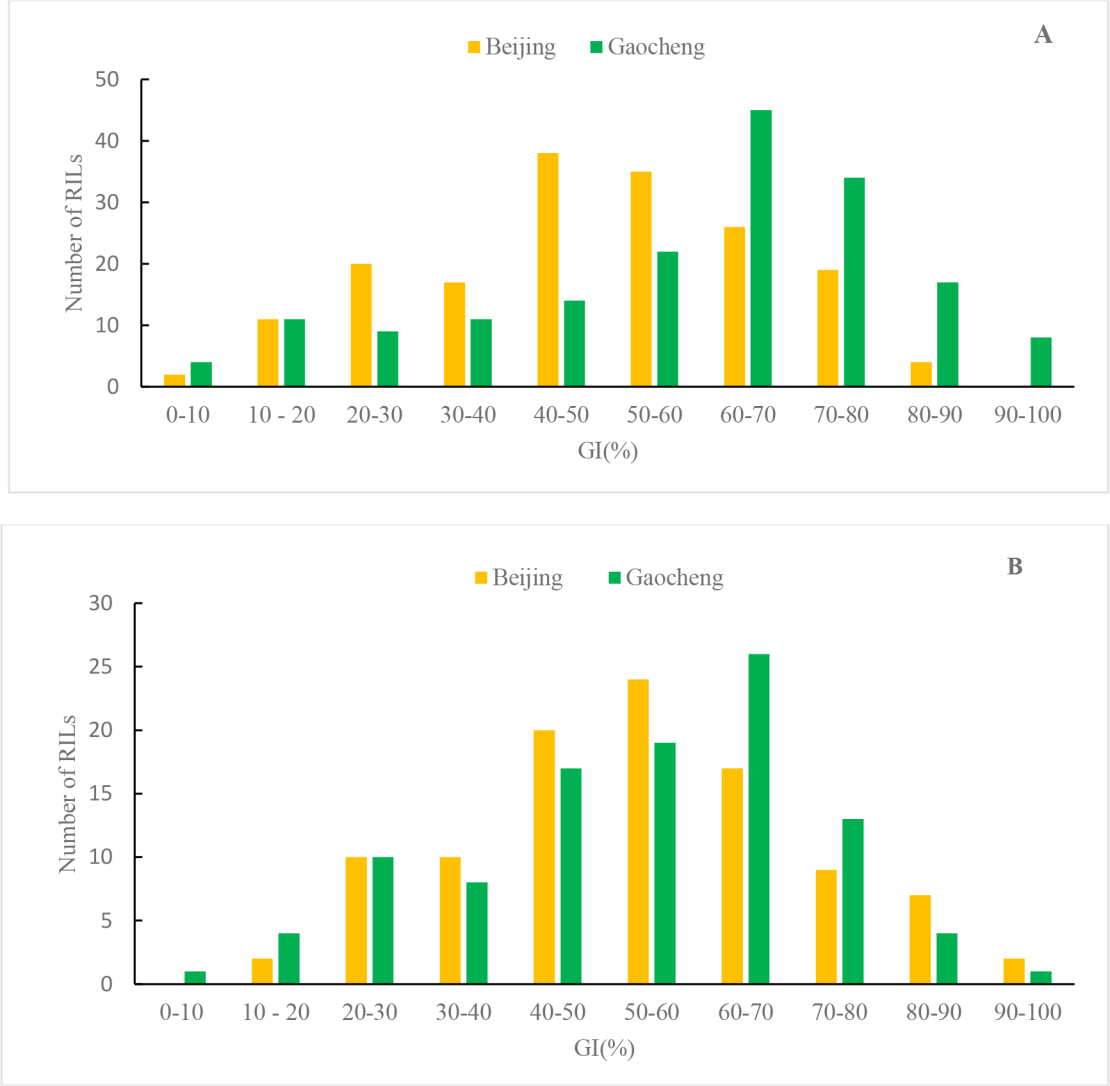
GI frequency distribution of WJ **(A)** and WZ **(B)** RLs in two environments.

The GI frequency distributions of both RIL populations in Beijing and Gaocheng exhibited nearly normal distribution (Beijing) or negatively skewed distribution(Gaocheng), with a significant increase in the number of families tending toward PHS susceptibility (**Figure 2**). This pattern suggests that the genes governing PHS resistance in the parents display quantitative trait inheritance.

Analysis of variance revealed that the environment had a highly significant effect on the GI of the WJ RIL population (F = 25.019, *P* < 0.001), whereas no significant environmental difference was observed on the GI of the WZ RIL population (**Table 4**).

**Table 4:** Variance analysis for GI of the two RIL populations.

RILs	Environment (Mean ± SD)		*F*	*P*
WJ	Beijing (n=176)	Gaocheng (n=176)	25.019	0.000**
	48.55 ± 18.69	59.47 ± 21.59		
WZ	Beijing (n=103)	Gaocheng(n=103)	0.022	0.881
	53.59 ± 17.81	53.21 ± 17.96		

**P < 0.01.

### 3.3 Allelic variation of *TaMFT-3A* and *TaMKK3-4A* in the two RIL populations

**Figure 3:**
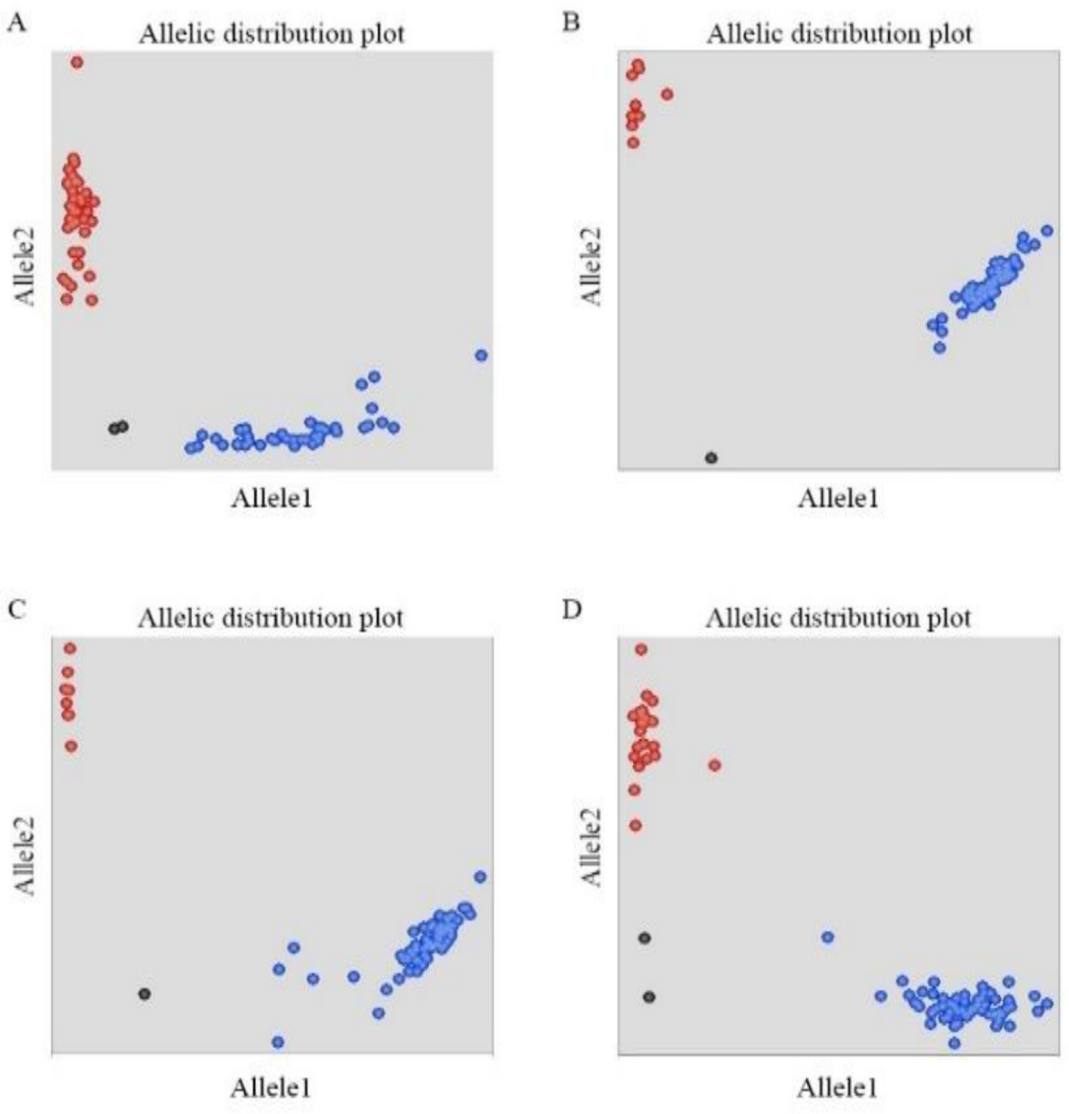
Illustration of KASP assays of *TaMFT-3A* and *TaMKK3-4A* in two RILs **(A)** SNP -222 genotyping, **(B)** SNP +646 genotyping, **(C)** SNP +666 genotyping, **(D)** SNP +660 genotyping. The blue and red dots represent the FAM and HEX labelled alleles, respectively. The black dots represent the negative control (ddH_2_O).

KASP markers were used to detect the genotypes of *TaMFT-3A* and *TaMKK3-4A* to determine the distribution frequencies of the different SNP loci in the two RIL populations (**Figure 3**). For the –222 locus of the *TaMFT-3A* gene in the WJ RIL population, the frequencies of the allelic variants C (resistance) and T (susceptibility) were 48.86% and 51.14%, respectively. At the +646 locus, the frequencies of the allelic variants G (resistance) and A (susceptibility) were 92.61% and 7.39%, respectively. For the +666 locus, the frequencies of the allelic variants T (resistance) and A (susceptibility) were 92.05% and 7.95%, respectively. For the +660 locus of the *TaMKK3-4A* gene, the frequencies of the allelic variants G (resistance) and T (susceptibility) were 62.50% and 37.50%, respectively (**Table 5**).

**Table 5:** Allele frequencies of *TaMFT-3A* and *TaMKK3-4A* in the WJ and WZ RIL populations.

RILs	Gene	Allele	Frequency (%)	GI (%)	
				Gaocheng	Beijing
WJ	*TaMFT-3A*	-222C	48.86	57.84 ± 22.62^a^	44.44 ± 19.50^a^
		-222T	51.14	61.05 ± 20.97^a^	52.7 ± 16.95^c^
		+646G	92.61	59.07 ± 17.30^a^	47.48 ± 15.07^a^
		+646A	7.39	64.95 ± 17.46^a^	64.47 ± 10.07 ^c^
		+666T	92.05	59.25 ± 17.17^a^	47.59 ± 15.14^a^
		+666A	7.95	64.24 ± 19.70 ^a^	62.86 ± 10.89 ^c^
	*TaMKK3-4A*	-snp1G	62.50	62.44 ± 20.83^a^	49.17 ± 19.41^a^
		-snp1T	37.5	54.26 ± 22.38^b^	47.07 ± 17.61^a^
WZ	*TaMFT-3A*	-222C	58.25	53.75 ± 17.69^a^	46.93 ± 15.92^a^
		-222T	41.75	51.89 ± 19.70^a^	62.57 ± 16.37^c^
		+646G	90.29	53.12 ± 15.64^a^	52.08 ± 13.41^a^
		+646A	9.71	51.50 ± 8.98^a^	68.27 ± 15.72^c^
		+666T	91.26	52.97 ± 15.71^a^	52.12 ± 13.66^a^
		+666A	8.74	52.87 ± 7.35^a^	68.62 ± 14.02^c^
	*TaMKK3-4A*	-snp1G	72.55	51.98 ± 18.62^a^	55.45 ± 17.23^a^
		-snp1T	27.45	55.95 ± 18.47^a^	49.77 ± 18.21^a^

^a^no significant difference, ^b^significant difference at *P* < 0.05, ^c^highly significant difference at *P* < 0.01.

In the WZ RIL population, for the –222 locus of the *TaMFT-3A* gene, the frequencies of the allelic variants C (resistance) and T (susceptibility) were 58.25% and 41.75%, respectively. At the +646 locus, the frequencies of the allelic variants G (resistance) and A (susceptibility) were 90.29% and 9.71%, respectively. For the +666 locus, the frequencies of the allelic variants T (resistance) and A (susceptibility) were 91.26% and 8.74%, respectively. For the +660 locus of the *TaMKK3-4A* gene, the frequencies of the allelic variants G (resistance) and T (susceptibility) were 72.55% and 27.45%, respectively (**Table 5**).

### 3.4 Evaluation of the effect of allelic variations of *TaMFT-3A* and *TaMKK3-4A* genes on PHS resistance

The *t*-test analysis of the GI values of the RILs carrying the *TaMFT-3A* SNP alleles of resistance and susceptibility revealed that the allelic variation at positions –222, +646, and +666 were highly correlated with PHS trait. In the two RIL populations, the allelic variations at the positions –222 (C and T), +646 (G and A), and +666 (T and A) of the *TaMFT-3A* gene showed extremely significant differences in the GI values in Beijing, but no significant differences were detected in Gaocheng (**Table 5**).

The allelic variation at position +660 of the *TaMKK3-4A* gene exhibited no correlation with PHS resistance. In the WJ RIL population, no significant differences in GI values were observed among RILs carrying allelic variations at position +660 (G and T) in the Beijing site, but a significant negative correlation was detected in the Gaocheng site. In the WZ RIL population, no significant differences in GI values were observed among RILs carrying different allelic variations at position +660 (G and T) in either Beijing site or Gaocheng site (**Table 5**).

Above results indicate that the effects of allelic variations in *TaMFT-3A* and *TaMKK3-4A* on PHS resistance are distinct, with the impact being influenced by genetic background and environmental factors.

### 3.5 The effect of allelic variation combination of *TaMFT-3A* and *TaMKK3-4A* genes on PHS resistance

Using the GI of the allelic variation combination of *TaMFT-3A* (–222C, +646G, and +666T) and *TaMKK3-4A* (+660G) (CGTG) as a control, it was observed that the effect of different allelic variation combinations on PHS resistance was influenced by genetic background and environmental conditions.

In the WJ RIL population, the combined allelic variation TAAG exhibited a significant difference compared to the combination of CGTG in the Beijing site but showed no significant differences in the Gaocheng site. The combined allelic variation of TGTT and CGTT did not significantly differ from the combination of CGTG in either Beijing or Gaocheng sites (**Table 6**). In the WZ RIL population, the combined allelic variation TAAG showed a significant difference from that of CGTG in the Beijing site (*P* < 0.01), but no significant difference in Gaocheng site. The combined allelic variation TGTG was also significantly different from that of CGTG in the Beijing site, whereas no significant difference was observed in Gaocheng site. The combined allelic variation of TGTT and CGTT did not exhibit significant differences from that of CGTG in either Beijing or Gaocheng sites (**Table 6**). Above that, the results suggest that in both RIL populations, the PHS trait exhibited significant changes only when the three loci of *TaMFT-3A* (–222, +646, and +666) collectively are formed to express the susceptible TAA combination. In contrast, haplotype variation at the +660 locus of *TaMKK3-4A* appeared to have no effect on PHS resistance trait.

**Table 6:** Pyramiding effect of *TaMFT-3A* and *TaMKK3-4A* alleles on PHS resistance.

Materials	*TaMFT-3A*			*TaMKK3-4A*	Frequency (%)	GI (%)	
	-222	+646	+666	+660		Gaocheng	Beijing
W/J	C	G	*T*	*G*	39.62	60.30 ± 21.76 ^a^	46.42 ± 19.00 ^a^
	T	A	*A*	*G*	7.55	70.03 ± 19.90^a^	64.47 ± 11.81^b^
	T	G	*T*	T	19.81	53.56 ± 21.91^a^	49.50 ± 14.31^a^
	C	G	*T*	T	33.02	54.29 ± 22.43^a^	46.59 ± 18.02^a^
W/Z	C	G	*T*	*G*	40.59	52.90 ± 19.19 ^a^	47.94 ± 15.68 ^a^
	T	A	*A*	*G*	8.91	52.87 ± 8.99 ^a^	68.62 ± 17.18 ^c^
	T	G	*T*	G	24.75	49.90 ± 20.55 ^a^	63.29 ± 12.84 ^c^
	T	G	*T*	T	7.92	58.60 ± 25.71 ^a^	54.43 ± 21.17 ^a^
	C	G	*T*	T	17.82	56.11 ± 14.55 ^a^	46.20 ± 16.01 ^a^

^a^no significant difference; ^b^significant difference at *P* < 0.05; ^c^highly significant difference at *P* < 0.01.

## 4 Discussion

Currently, in Chinese wheat production, mechanical harvesting with direct threshing is widely adopted, and the required grain moisture content is typically maintained below 16%. However, if the harvest is delayed and rainy weather occurs during maturity, the likelihood of PHS disasters increases dramatically. PHS is a complex biological trait controlled not only by seed dormancy (Shingo et al., 2011; Torada et al., 2016) but also by factors such as seed coat color (Lang et al., 2021), glume and awn characteristics, physiological status (Sun et al., 2003; Barua et al., 2012), and environmental conditions, including humidity, temperature, and sunlight after flowering (Tai et al., 2021).

Results from this study demonstrate that crosses derived from PHS-resistant and PHS-susceptible parents can produce RIL offspring with enhanced PHS resistance. In conventional wheat breeding programs, spike selection is typically emphasized during the F_2_ and F_3_ generations, while individual plants exhibiting superior agronomic traits are selected from spike rows in the F_4_ generation. Thus, selection for PHS resistance can feasibly begin in the F_4_ or F_5_ generation, when lines display relatively stable heredity and favorable agronomic performance. Moreover, PHS resistance is also affected by the physiological state of the seeds, failure to group and test materials with different maturity periods during the early generations may inadvertently lead to a shift toward late maturity (Sun et al., 2010; Xiao et al., 2004).

The germination ability of mature seeds is affected by numerous physiological and environmental factors during seed development, after-ripening, and germination. Therefore, when assessing PHS resistance, it is crucial to minimize interference from factors such as inconsistent physiological maturity, variable grain moisture content, and poor seed development. For example, wheat grains that develop under relatively humid conditions during the later stages of maturity can exhibit reduced dormancy (Mares, 1984). Many studies have shown that seed dormancy is correlated with geographical distribution, and environmental factors can cause differences in dormancy among seeds of the same variety from different locations and years (Andersson and Milberg, 1998; Gao et al., 2021; Schütz and Rave, 2003). Environmental conditions experienced during seed development, such as temperature and humidity, can affect hormone levels (Barua et al., 2012), trigger epigenetic modifications (Whittle et al., 2009), and influence gene interactions (Nakamura et al., 2011). Consequently, evaluating PHS resistance across multiple locations and years under different environmental conditions is essential for accurately assessing the resistance level of wheat germplasm.

*TaMFT-3A* and *TaMKK3-4A* are two cloned genes from white-grained wheat varieties that confer resistance to PHS, with allelic variations at different positions in these gene sequences being closely associated with the level of PHS resistance (Liu et al., 2015; Shorinola et al., 2017). This study further shows that allelic variations at the –222, +646, and +666 loci of the *TaMFT-3A* gene are highly correlated with PHS resistance, whereas the allelic variation at the +660 locus of the *TaMKK3-4A* gene exhibits no correlation that is also influenced by environmental conditions. Given the complex interaction between genes and the environment, achieving robust PHS resistance in wheat cannot rely solely on a single resistance gene. These findings are consistent with recent studies (Wang et al., 2020), which indicate the CGA combination at the –222, +646, and +666 positions of *TaMFT-3A* confers the highest level of PHS resistance, while the TAT combination is associated with the poorest resistance. For the *TaMKK3-4A* gene, Lin et al. (2018) reported that its presence in N/T and N/A BC_2_F_2_ populations did not significantly reduce the germination rate, with its effectiveness being affected by environmental factors. Similarly, Vetch et al. (2022) found no association between allelic variation at the *TaMKK3-4A* locus and PHS resistance in a hard red winter wheat doubled haploid population from North America, and Huang et al. (2022) observed no significant differences in the GI among different allelic variations at the *TaMKK3-4A* locus in 326 Chinese winter wheat varieties.

Regarding the pyramiding effect of *TaMFT-3A* and *TaMKK3-4A*, this study indicates that the allelic combination at the –222, +646, and +666 loci of *TaMFT-3A* plays a predominant role in determining PHS resistance. When this combination is TAA, PHS resistance is significantly reduced, and there is no significant correlation with the haplotype variation at the +660 locus of *TaMKK3-4A*. Although Lin et al. (2018) suggested that *TaMFT-3A* and *TaMKK3-4A* exert an additive effect on PHS resistance, our findings imply that this additive effect is modulated by environmental conditions during seed maturation and is more pronounced under controlled greenhouse conditions than in the field. Given that PHS resistance in wheat is regulated by multiple genes, conventional breeding alone is insufficient for developing resistant varieties. Assessing the effects of PHS resistance gene loci under various genetic backgrounds and environmental conditions will facilitate the effective utilization of resistant germplasm and improve the efficiency of genetic enhancement of PHS resistance through MAS.

## 5 Conclusion

This study evaluated the effects of different allelic variations and their combinations in the *TaMFT-3A* and *TaMKK3-4A* genes on PHS resistance in two RIL populations, WZ and WJ, across two environments. The results indicate that PHS resistance is influenced not only by the genetic background but also by environmental factors during seed development. The highest level of PHS resistance was observed when the –222, +646, and +666 loci of the *TaMFT-3A* gene were in the CGA combination, whereas the TAA combination was associated with increased susceptibility to PHS. In contrast, the haplotype variation at the +660 locus of the *TaMKK3-4A* gene showed no correlation with PHS resistance and was subject to environmental influences. These findings provide a theoretical basis for the rational utilization of these PHS resistance genes in molecular breeding efforts aimed at enhancing wheat resilience to PHS.

## Data Availability

The datasets presented in this study can be found in online repositories. The names of the repository/repositories and accession number(s) can be found in the article/**Supplementary Material**.
