# Supplementary material for: Evaluation of TaMFT-3A and TaMKK3-4A alleles on wheat pre-harvest sprouting
Source: Front Plant Sci. 2025 May 14;16:1594385. doi: 10.3389/fpls.2025.1594385 (PMC12116531; doi:10.3389/fpls.2025.1594385)
Supplement: Supplementary file 1 [file Table1.docx]

**Supplementary Table S1** Environmental statistics of field experiments conducted in Beijing and Gaocheng, Hebei, China in 2020-2021 growing season.

| **Location** | **Max. Temp. (℃)** | **Min. Temp. (℃)** |
| --- | --- | --- |
| Beijing | 26.96±4.42 | 16.55±3.20 |
| Gaocheng | 29.49±3.53 | 17.9±3.14 |

Temperature range are calculated from May 1 to June 15 in 2021. Data is from https://lishi.tianqi.com.
